# The Braveheart amphipod: a review of responses of invasive *Dikerogammarus villosus* to predation signals

**DOI:** 10.7717/peerj.5311

**Published:** 2018-08-02

**Authors:** Łukasz Jermacz, Jarosław Kobak

**Affiliations:** Department of Invertebrate Zoology, Faculty of Biology and Environmental Protection, Nicolaus Copernicus University, Torun, Poland

**Keywords:** Predator consumptive and non-consumptive effects, Prey–predator interaction, Invasive species, Anti-predator strategy, Kairomones

## Abstract

Predator pressure is a fundamental force driving changes at all levels of the community structure. It may protect native ecosystems from alien species. Therefore, resistance to diverse predators resulting from a universal anti-predator strategy seems crucial for invasion success. We present a comprehensive review of the responses of an invasive amphipod *Dikerogammarus villosus* to sympatric and allopatric predator signals. We summarize diverse aspects of the gammarid anti-predator strategy, including predator identification, morphological and behavioural adaptations, effectiveness of shelter use and resistance to indirect predator effects. The response of *D. villosus* is independent of predator species (including totally allopatric taxa), which assures the high flexibility of its predator recognition system. It has a harder exoskeleton and better capability of utilizing shelters compared to other gammarids, resulting in relatively high resistance to predators. Therefore, it can use predator kairomones as indirect food signals (sharing the diet with the predator) and follow the predator scent. This resistance may allow *D. villosus* to reduce the costs of its physiological responses to predators and sustain growth in their presence. This might facilitate invasion success by increasing its competitive advantage.

## Introduction

*Dikerogammarus villosus* (Sovinsky, 1894) is a gammarid of Ponto-Caspian origin, commonly regarded as one of the most invasive freshwater species in the world (DAISIE, 2009). In its native region, it lives in the lower courses of large rivers of the Black, Azov and Caspian Sea basins, and in limans formed at their outlets (Rewicz et al., 2014). It has spread in central and western Europe using the southern migration corridor through the Danube and Rhine rivers (Bij de Vaate et al., 2002), as well as the central corridor, through the Dnieper, Bug, Vistula and Elbe rivers (Grabowski, Bacela & Konopacka, 2007; Mastitsky & Makarevich, 2007). At present, it occupies the widest novel range (most of Europe, excluding the Iberian and Scandinavian Peninsulas, but including Great Britain) and reaches the highest abundances in invaded areas within the group of several invasive Ponto-Caspian gammarid species (Rewicz et al., 2014; Rewicz et al., 2017; Šidagyte et al., 2017; Gusev, Guseva & Sudnik, 2017).

In novel areas, *D. villosus* exerts a strong impact on local biota through several mechanisms (Gergs & Rothhaupt, 2015). Firstly, it is an omnivore with a tendency to animal food, efficiently preying on many invertebrate species (Krisp & Maier, 2005), including intra-guild predation on local amphipod species (Dick & Platvoet, 2000; MacNeil & Platvoet, 2005; Kinzler et al., 2009). *D. villosus* strongly prefers animal food over plants (Van Riel et al., 2006; Gergs & Rothhaupt, 2008b) and grows better on it (Gergs & Rothhaupt, 2008a). However, recent field studies have revealed that it can also act as a typical herbivore, consuming mainly plant food (Maazouzi et al., 2009; Hellmann et al., 2015; Koester, Bayer & Gergs, 2016). This points to its high plasticity and ability to use various available food resources (Mayer, Maas & Waloszek, 2012), depending on local circumstances, such as the community composition (Hellmann et al., 2017). Moreover, it efficiently competes with other gammarids, both native and other aliens, for food, shelters and optimum habitats (Dick, Platvoet & Kelly, 2002; Hesselschwerdt, Necker & Wantzen, 2008; Jermacz et al., 2015a). Competitive tensions and intra-guild predation are responsible for the reduction in the occupied ranges and abundances of native species, which are being outcompeted to less suitable habitats and sometimes even locally displaced (Dick, Platvoet & Kelly, 2002; Muskó et al., 2007; Hesselschwerdt, Necker & Wantzen, 2008; Platvoet et al., 2009). Furthermore, *D. villosus* can exert some more subtle effects on ecosystem functioning. For instance, being a less efficient shredder than other amphipods, displaced by its appearance, *D. villosus* may negatively affect food webs by reducing the numbers of organisms relying on shredded organic material (MacNeil et al., 2011). On the other hand, *D. villosus* does not respond to predation risk by reduction in feeding (Jermacz & Kobak, 2017); therefore, it can still be capable of shredding organic material when other amphipods suffer non-consumptive costs of predator pressure (Abjörnsson et al., 2000; Jermacz & Kobak, 2017; Richter et al., 2018).

The invasion success of *D. villosus* is regarded to result from several traits of its biology, including its fast growth rate, high fecundity, tolerance to wide ranges of abiotic factors, in particular raised salinity (Devin & Beisel, 2007), as well as high plasticity and omnivory (as reviewed by Grabowski, Bacela & Konopacka, 2007). Another trait contributing to its spread is the ability to adhere to various hard substrata and artificial materials, including boat hulls and diving equipment (Bacela-Spychalska et al., 2013). Although *D. villosus* is not well adapted to air exposure (Poznańska et al., 2013), under suitable conditions, e.g., hidden in a zebra mussel colony, individuals of this species can survive several days without water (Martens & Grabow, 2008), sufficient for successful transport to another water body.

Yet another important trait which can potentially affect the invasive potential of the species is an effective strategy of energy saving (Becker et al., 2016) observed also under predation risk (Jermacz et al., 2017; Jermacz & Kobak, 2017). In recent years, we have conducted a series of experimental studies on the reactions of *D. villosus* to predation cues and their potential implications for its functioning and invasiveness. In this review, we provide a synthesis of our research on these topics accompanied by the results of other authors on the biology of *D. villosus* and related amphipod species.

### Survey methodology

To obtain a comprehensive set of literature reports on interactions between predators and amphipod prey, we conducted a literature survey in the Scopus database, using the following keywords: *D. villosus* or gammarid or amphipod combined with the following: anti-predator response or predator impact or anti-predator behaviour or predator defence or predator kairomone or predation risk or prey response. We found 67, 115 and 927 articles, respectively, meeting the criteria, 11 of which were of our own authorship. Among these articles were chosen key papers related to defence strategies exhibited by native and invasive amphipods.

### Prey–predator relationships in the context of biological invasions

Predation is one of the most powerful forces in nature, affecting the evolution of prey and predator species and modifying interactions among organisms (Mowles, Rundle & Cotton, 2011; Turner & Peacor, 2012). On the one hand, predators kill and consume prey individuals, removing them from the population and creating selective pressure, which results in so called “consumptive effects” of a predator (Werner & Peacor, 2003). On the other hand, prey species respond to the presence of predators by various forms of constitutive (permanent) and induced defences, stimulated by the presence of a predator. These defence mechanisms include behavioural (De Meester et al., 1999; Gliwicz, 2005), morphological (Pettersson, Nilsson & Brönmark, 2000; Dzialowski et al., 2003; James & McClintock, 2017), physiological (Slos & Stoks, 2008; Glazier et al., 2011) and life-history related (Ślusarczyk, Dawidowicz & Rygielska, 2005) changes aiming at reducing the probability and/or efficiency of a predator attack. Defence responses are displayed by a wide range of taxa, from protozoans (Wiackowski, Fyda & Ciećko, 2004) through virtually all invertebrate taxa (Koperski, 1997; Lass & Spaak, 2003; Thoms et al., 2007; Kobak, Kakareko & Poznańska, 2010) to vertebrates (Gliwicz, 2005). Anti-predation mechanisms can be quite costly, consuming energy designated for the construction of defensive structures and compromising the habitat quality and/or food abundance, which finally leads to the decrease in growth rate and reproduction (Gliwicz, 1994; Gliwicz, 2005; De Meester et al., 1999; Clinchy, Sheriff & Zanette, 2013). These energetic expenses are called “non-consumptive effects” of predator presence (Werner & Peacor, 2003) and sometimes generate losses comparable to those caused by consumptive predator effects (Preisser, Bolnick & Benard, 2005; Creel & Christianson, 2008). Therefore, the ability to adequately recognize the danger imposed by predators, depending on their feeding mode (Wudkevich et al., 1997; Wooster, 1998; Abjörnsson et al., 2000), present condition (e.g., satiation level) (Abjörnsson et al., 1997), abundance (Pennuto & Keppler, 2008) and size (Kobak, Kakareko & Poznańska, 2010) is crucial for avoiding unnecessary (leading to energy wasting) or maladaptive (increasing the probability of death) responses.

Biological invasions add a new and interesting aspect to predator–prey interactions. In old systems, where predator and prey coevolve together for a long time, both are well adjusted to each other. The responses of prey species can be fine-tuned to specific predators (Wudkevich et al., 1997; Weber, 2003; Boeing, Ramcharan & Riessen, 2006), but also predator preying modes allow them to feed efficiently on available victims (Gliwicz, 2005). However, alien species, just transported to their novel locations, face completely new, unknown communities, containing new predators and new prey. In accordance with the Enemy Release Hypothesis (Torchin et al., 2003), as a consequence of the loss of natural parasites and predators, introduced species suffer lower pressure than native species. At the same time, local consumers may be unfamiliar with alien prey and unable to forage on them efficiently (Meijer et al., 2016). On the other hand, alien species are also not adapted to their new, potential predators which may prevent them from employing efficient anti-predation mechanisms and lead to an evolutionary trap: inefficient or even maladaptive responses or the lack of reactions to a danger (Salo et al., 2007; Zuharah & Lester, 2010).

Recognition of a predator may be based on variable stimuli, including chemical, visual and/or mechanical cues. In the aquatic environment, due to its relative darkness and high density of the medium, chemical recognition is regarded as the most important (Brönmark & Hansson, 2000). This general rule also applies to gammarids detecting their predators (Wisenden et al., 2009; Hesselschwerdt et al., 2009; Jermacz, Dzierżyńska-Białończyk & Kobak, 2017). Prey organisms can potentially recognize three sources of chemical predation cues: alarm cues produced by wounded conspecifics (Czarnołeski et al., 2010; Kobak & Ryńska, 2014; Jermacz, Dzierżyńska-Białończyk & Kobak, 2017), scents of consumed conspecifics included in predator faeces (Ślusarczyk & Rygielska, 2004; Jermacz, Dzierżyńska-Białończyk & Kobak, 2017) or other exudates and/or direct predator metabolites, independent of their diet (Kobak, Kakareko & Poznańska, 2010; Jermacz, Dzierżyńska-Białończyk & Kobak, 2017). The first two options can be potentially utilized by alien organisms to detect unknown predators. Moreover, alien organisms can recognize predators taxonomically related to those living in their native range (Sih et al., 2010) or use learning to associate new predator scents with the perceived danger cues (Chivers, Wisenden & Smith, 1996; Wisenden, Chivers & Smith, 1997; Martin, 2014). The latter approach is commonly exhibited by fish (Korpi & Wisenden, 2001), whereas in invertebrates predator recognition is often innate, displayed also by naïve individuals (Dalesman, Rundle & Cotton, 2007; Ueshima & Yusa, 2015).

### Predator recognition by *D. villosus*

For a perfect invasive species, the mechanism of predator detection should be universal, enabling the recognition and subsequent response to a novel predator without a common evolutionary history. As a consequence of an improper identification of a predator signal, prey species are exposed to higher predation due to the lack of responses or maladaptive responses (Abjörnsson, Hansson & Brönmark, 2004; Banks & Dickman, 2007). Such a scenario was presented by Pennuto & Keppler (2008) who demonstrated that a native *Gammarus fasciatus* is able to avoid a narrower range of potential predators than an invasive *Echinogammarus ischnus*. Moreover, ineffective recognition of danger could result in costly defence reactions when the predation risk is low (Lima & Dill, 1990; Dunn, Dick & Hatcher, 2008) as was experimentally shown for *Gammarus minus* responding to a predatory fish *Luxilus chrysocephalus* (Wooster, 1998). Therefore, appropriate predation risk assessment is crucial for an adequate response and optimization of energy expenditure.

Laboratory experiments demonstrated the ability of *D. villosus* to recognize diverse fish predators, including bottom dwellers: the racer goby *Babka gymnotrachelus* (Jermacz et al., 2017; Jermacz, Dzierżyńska-Białończyk & Kobak, 2017), European bullhead *Cottus gobio* (Sornom et al., 2012) and spiny-cheek crayfish *Orconectes limosus* (Hesselschwerdt et al., 2009), as well as fish swimming in the water column: the Eurasian perch *Perca fluviatilis*, Amur sleeper *Perccottus glenii* (Ł Jermacz & J Kobak, pers. obs., 2015–2016) and red-bellied piranha *Pygocentrus nattereri* (Jermacz, Dzierżyńska-Białończyk & Kobak, 2017). Among these species, the goby, bullhead and perch have co-occurred with the gammarid in its home range, the Amur sleeper and crayfish were met several dozen years ago in its novel areas, whereas the piranha had no previous contact with *D. villosus*. The above-mentioned studies indicate a universal method of predator recognition exhibited by *D. villosus,* effective with regard to both native and novel predatory species. A situation when potential naïve prey recognizes and responds to a novel predator can be explained by several mechanisms. For example, conspecifics can be present in the predator diet, providing information about predation risk (Chivers & Smith, 1998), as was demonstrated for another invasive gammarid *Pontogammarus robustoides* (Jermacz, Dzierżyńska-Białończyk & Kobak, 2017). Moreover, the novel predator can be closely related to some native predators (Ferrari et al., 2007; Sih et al., 2010) and therefore release similar signals.

The avoidance reactions of *D. villosus* were studied by Jermacz, Dzierżyńska-Białończyk & Kobak (2017) in a flow-through Y-maze allowing gammarids to select an arm with or without the scent of predators fed on different diets. This study indicated that the avoidance of predators was induced in the presence of kairomones emitted by hungry predators (starving for 3 days), including totally allopatric, tropical *P. nattereri* (Jermacz, Dzierżyńska-Białończyk & Kobak, 2017). The avoidance response of *D. villosus* to another hungry predator, American spiny-cheek crayfish, was noted by Hesselschwerdt et al. (2009). Thus, the predator identification system of *D. villosus* seems to be independent of the presence of conspecifics in the predator’s diet. Nevertheless, it should be noted that *D. villosus* did also recognize the predator diet and used it as an additional source of information about the predator status and current level of predation risk, though its responses to satiated predators did not include avoidance (see the chapter “Positive response of *D. villosus* to the predation cue” below) (Jermacz, Dzierżyńska-Białończyk & Kobak, 2017). Avoidance of a hungry predator, which is most determined to obtain food, and modifications of the responses to satiated predators suggest that *D. villosus* is capable of effective risk assessment and flexible responses, adjusted to the current situation. A similar relationship between the level of predator satiation and prey response was observed in the case of a water beetle *Acilius sulcatus*, responding only to hungry perch, but not to satiated fish (Abjörnsson et al., 1997).

The versatility of the predator detection mechanism of *D. villosus* could be related to the fact that active components of kairomones emitted by unrelated predators are often very similar (Von Elert & Pohnert, 2000). Therefore prey can react to diverse predators, including those which evolved in isolated ecosystems. In temperate European water bodies, fish usually have broad diet ranges and most of them feed on invertebrate food at least at particular life stages (Wootton, 1990; Gerking, 1994). Thus, a general response to hungry fish of particular size seems beneficial under such conditions. *D. villosus* is an invasive species characterized by a high dispersal rate. During the dispersal, the probability of meeting a novel predator is high, therefore species exhibiting universal defence mechanisms and/or the capability of quick adaptations are more likely to be successful invaders.

### Anti-predator defence mechanisms of *D. villosus*

#### Site selection and shelter occupancy

For a benthic organism, one of the most important elements of the anti-predator strategy is related to the optimal substratum choice. In general, prey survival rate increases with the level of substratum complexity and heterogeneity (Crowder & Cooper, 1982; Holomuzki & Hoyle, 1988; Czarnecka, 2016). Therefore, the distribution of benthic invertebrates depends on the bottom character (Czarnecka et al., 2009; Jermacz et al., 2015b) and their efficiency in using available substrata as shelters (Holomuzki & Hoyle, 1988; Kobak, Jermacz & Płąchocki, 2014; Kobak et al., 2016).

Compared to other gammarids, *D. villosus* is regarded as a sit-and-wait animal, spending most of its time in a shelter (Kinzler & Maier, 2006; Kley et al., 2009; Platvoet et al., 2009; Beggel et al., 2016). It prefers the substratum consisting of large gravel or stones (>6 cm in diameter), which provides it with suitable protection and enough empty space to move (Kley et al., 2009; Boets et al., 2010; Kobak, Jermacz & Dzierżyńska-Białończyk, 2015). Perhaps due to its low activity (Van Riel et al., 2007; Beggel et al., 2016), changes in shelter occupancy in the presence of predators observed in various studies are ambiguous. In the presence of benthivorous fish (European bullhead), *D. villosus* was observed to reduce its presence in the open field (i.e., outside shelters) and activity considerably, from ca. 55% under control conditions to only 20% of the total experimental time (Sornom et al., 2012). However, in other studies, the reduction in open field occupancy or activity in the presence of predatory fish was only slight, though significant (Beggel et al., 2016; Jermacz et al., 2015a; Jermacz & Kobak, 2017), or no response was observed at all Jermacz et al. (2017). These discrepancies might have resulted from the varying quality of shelters that could be occupied always or only in the presence of danger, as well as from the location of food. Sornom et al. (2012) found that in the presence of predators *D. villosus* decreased its activity and stayed more often in shelters made of holes in the solid substratum, whereas mesh shelters were always occupied irrespective of predator presence (>80% of the total time). In the studies by Jermacz et al. (2015a) and Jermacz & Kobak (2017) gammarids spent more than 95% of the total experimental time in gravel substratum under predator absence, which allowed for only a small, though still significant change in response to predators. Jermacz et al. (2017) and Beggel et al. (2016) found that gammarids spent most of their time in gravel shelters even in the absence of predators. Jermacz & Kobak (2017) observed gammarids to limit their occupation of the open space in the presence of predators when food was present in the direct vicinity of their shelters, whereas they kept exploring the unsheltered area in search of distant food sources. Thus, the presence of food can increase gammarid activity, which in turn can be reduced by the predation cue when the food is available at a short distance.

*D. villosus* often occurs on hard and complex substrata, difficult to access by predators. Stone substratum was found to offer it more protection against fish predation compared to *Gammarus fossarum* and *G. pulex*, but this advantage disappeared on sand (Kinzler & Maier, 2006). In the wild, *D. villosus* was often found associated with zebra mussel (*Dreissena polymorpha*) colonies (e.g., Devin et al., 2003; Boets et al., 2010). Kobak, Jermacz & Płąchocki (2014) demonstrated that living dreissenids provided *D. villosus* with the most effective shelter against fish predators (the racer goby and Amur sleeper), compared to stones, macrophytes and shell litter. It should be noted that this shelter was also useful against a species without a common evolutionary history and exhibiting a different feeding strategy than the sympatric gobies (the Amur sleeper). This study demonstrated the positive effect of dreissenids on prey survival only in the case of *D. villosus*, but not for other invasive (*P. robustoides*) and native (*Gammarus fossarum*) species. However, in contrast to our studies, Beekey, McCabe & Marsden (2004) showed that also native prey, including amphipods, experiences lower predation pressure in dreissenid beds.

Dreissenid beds can offer more effective protection than other substrata (Kobak, Jermacz & Płąchocki, 2014), to less active species, such as *D. villosus* (Kobak et al., 2016), spending most time in the shelter (Beggel et al., 2016; Jermacz & Kobak, 2017). This indicates that the presence of gregarious bivalves may promote the establishment of *D. villosus*. Dreissenid colonies, in contrast to other substratum types, form aggregations of objects bound with one another by byssal threads, hard to penetrate by fish (Kobak, Kakareko & Poznańska, 2010) which, in association with the high attachment ability of *D. villosus* compared to other gammarids (Bacela-Spychalska, 2016) may make a mussel bed a perfect shelter for this species. Moreover, the hard substratum which supplies not only shelter and clinging opportunity, but also food resources, such as a colony of *D. polymorpha*, seems to be an optimal habitat for the invasive gammarids and may allow them to limit their exploration activity (Jermacz & Kobak, 2017). Mussels provide both effective anti-predator protection (Beekey, McCabe & Marsden, 2004; McCabe et al., 2006; Kobak, Jermacz & Płąchocki, 2014) and valuable food resources, such as organic-rich pseudofaeces and macroinvertebrate prey of increased abundance (Gergs & Rothhaupt, 2008b; Kobak et al., 2016).

When shelters are limited (e.g., on sandy unvegetated nearshore bottoms in the wild), *D. villosus* exhibits an avoidance response to the predator cue, as shown by Hesselschwerdt et al. (2009) and Jermacz, Dzierżyńska-Białończyk & Kobak (2017) in a Y-maze. This response was observed in the presence of hungry predators (starving for 3 days), likely to pose the highest danger to their potential prey. Thus, in the absence of suitable shelters and the presence of a direct danger, a temporary increase in activity and active avoidance seems to be an optimum response. In natural conditions, such a response is likely to result in leaving the predator area or finding the nearest shelter, after which the activity is reduced as the predation risk decreases.

#### Aggregation forming

Shelter choice depends not only on the substratum quality but also the presence or absence of conspecifics and heterospecifics gammarids (Jermacz et al., 2015a; Jermacz et al., 2017). Laboratory experiments showed that *D. villosus* preferred shelters occupied by conspecifics over empty shelters and conspecifics located apart from shelters (Jermacz et al., 2017). Moreover, *D. villosus* exhibited a preference for shelters inhabited by conspecifics over those occupied by heterospecifics gammarids (*P. robustoides*), thus forming single-species aggregations (Jermacz et al., 2017). Nevertheless, in the presence of predators, the selectivity of gammarids was reduced and they grouped alike with conspecifics and heterospecifics. The choice of the substratum already inhabited by other prey individuals is an example of aggregation behaviour combined with the benefits of sheltered conditions. The main advantage of the aggregation strategy is a reduction in the individual risk of predation (Hamilton, 1971). On the other hand, the weakness of this strategy is the facilitation of detection by a predator, especially by species using vision for prey detection (Ioannou & Krause, 2008). However, when gammarids are aggregated under sheltered conditions, their detection seems to be difficult.

Notwithstanding the protective role of gammarid aggregations against predators, *D. villosus* did not increase the intensity of its grouping in shelters in the presence of predators (Sornom et al., 2012; Jermacz et al., 2017), in contrast to its relative, *Pontogammarus robustoides* (Jermacz et al., 2017). However, also in contrast to *P. robustoides*, *D. villosus* exposed to predation cues formed conspecific aggregations in open places, in the absence of shelters (Jermacz et al., 2017). The effectiveness of such a response as a protection against predators was demonstrated under laboratory conditions in which the racer goby avoided aggregated prey and consumed it less efficiently than singletons (Jermacz et al., 2017). This may be a consequence of the aforementioned clinging abilities of *D. villosus* (Bacela-Spychalska et al., 2013) and/or the hardness of its exoskeleton (Błońska et al., 2015), which are greater than those of other gammarids, such as *G. fossarum* or *P. robustoides* (Bacela-Spychalska et al., 2013) and increase predator handling costs, thus contributing to the resistance of such aggregations against predators. On the other hand, the easiest prey for predators were single inactive individuals (Jermacz et al., 2017), indicating that this state should be avoided by gammarids seeking protection against predation.

#### Depth selection

For an aquatic organism, the choice of an appropriate habitat is also related to water depth. Fish predation pressure at shallow nearshore locations can be significantly lower than at deeper sites (Gliwicz, Soń & Szynkarczyk, 2006; Perez et al., 2009). An experiment conducted by Kobak et al. (2017) in a 1 m deep tank with a depth gradient demonstrated that *D. villosus* in the presence of the racer goby relocated from the deepest zone, occupied preferentially under safe conditions, to the shallower bottom. Moreover, it also climbed upwards along the vertical tank walls and attached near the water surface (Kobak et al., 2017). Gobies are bottom-dwelling predators, rarely swimming freely in the water column; therefore the escape to the water column seems to be an effective response against them (Pinchuk et al., 2003). Our experimental results are reflected in field observations made in Lake Balaton occupied by Ponto-Caspian Gobiidae (Ferincz et al., 2016), where *D. villosus* occurs mainly on the stones near the water surface (Muskó et al., 2007).

#### External factors affecting gammarid responses to predators

The responses of *D. villosus* to predator cues are modified by environmental pollution, such as increased heavy metal concentration (Sornom et al., 2012). The gammarids exposed to the solution of 500 µg of cadmium per litre of water were observed to hide less often and be more active than the control individuals. Moreover, they no longer changed their behaviour in response to the presence of predators (Sornom et al., 2012).

Yet another potential factor that can potentially affect prey responses to predators is the presence of parasites, modifying host activity and it refuge use (Lafferty & Morris, 1996; Perrot-Minnot, Kaldonski & Cézilly, 2007; Kaldonski et al., 2008). *D. villosus* in European waters is parasitized by a microsporidian *Cucumispora dikerogammari* (Bacela-Spychalska et al., 2012). This parasite was found to affect the behaviour of its host, making it more active (Bacela-Spychalska, Rigaud & Wattier, 2014), which could increase the detection probability and in consequence the survival chance of infected prey. Activity increase could potentially expose parasitized individuals to predator attacks and reduce their defence capabilities, though at present no evidence exists for that and further studies are needed on this topic.

Finally, it should be noted that not all responses of *D. villosus* to predators can be considered as anti-predator defences (Table 1). The predator diet can strongly modify the behaviour of gammarids and switch their responses from typical avoidance to even preference for predator scents (Jermacz, Dzierżyńska-Białończyk & Kobak, 2017). See the chapter “Positive response of *D. villosus* to the predation cue” below for the details.

**Table 1:** Anti-predation mechanisms of *D. villosus* and other changes induced by the presence of predators.

Trait	Comments	References
Constitutive traits (not changing in the presence of predators, but potentially protective)		
Staying inactive in the shelter	The species is less active than other gammarids	Kley et al. (2009), Beggel et al. (2016) and Kobak, Rachalewski & Bacela-Spychalska (2016)
Aggregation in shelters	No increase in the presence of predators, but can contribute to the anti-predator protection	Sornom et al. (2012), Jermacz et al. (2017) and Jermacz, Dzierżyńska-Białończyk & Kobak (2017)
Hard exoskeleton	Compared to other gammarids	Błońska et al. (2015)
High clinging ability	Potentially may facilitate forming aggregations resistant to predators	Bacela-Spychalska (2016)
Changes induced by predators		
Increase in shelter occupancy time	Ambiguous results:	
	Shown in hole shelters, not shown in mesh shelters	Sornom et al. (2012)
	Shown in the vicinity of food, not shown when food was distant	Jermacz & Kobak (2017)
	Weak but significant effect	Jermacz et al. (2015a), Jermacz et al. (2015b) and Beggel et al. (2016)
Utilization of coarse substrata (stones or zebra mussel colonies) as shelters	More efficient compared to other gammarid species	Kinzler & Maier (2006), Kobak, Jermacz & Płąchocki (2014)
Active defence	Better survival than that of *G. fossarum* in the presence of fish and without shelters	Błońska et al. (2016)
Activity reduction	Shown in the presence of hole shelters, but not with mesh shelters	Sornom et al. (2012)
Active avoidance	The scents of hungry predators (crayfish and fish), starving for 3 days, in a Y maze	Hesselschwerdt et al. (2009), Jermacz et al. (2017) and Jermacz, Dzierżyńska-Białończyk & Kobak (2017)
Active preference	The scents of predators fed with conspecifics, other gammarids or chironomid larvae in a Y maze	Jermacz et al. (2017) and Jermacz, Dzierżyńska-Białończyk & Kobak (2017)
Selection of shallower depth	In a 1-m depth gradient, in the presence of a benthic predator	Kobak et al. (2017)
Aggregation in the open field		Jermacz et al. (2017) and Jermacz, Dzierżyńska-Białończyk & Kobak (2017)
Reduction in selectivity towards conspecifics	Gammarids stop preferring conspecifics and form groups independent of species	Jermacz et al. (2017) and Jermacz, Dzierżyńska-Białończyk & Kobak (2017)
Reduced consumption of food	Shown when food had to be searched for, not shown when food was present directly in the shelter	Jermacz & Kobak (2017)

#### D. villosus as prey

Prey selection is a universal process, in which predators must choose among prey that differ in density and defence strategy. To optimize their fitness, predators should select those prey species whose abundance is high and hunting cost is low (Emlen, 1966). Many variables can influence prey choice. Some of them are related to prey characteristics such as prey defence mechanisms, including behavioural (Andersson et al., 1986), morphological (Bollache et al., 2006), and physiological adaptations (Clinchy, Sheriff & Zanette, 2013) or to environmental factors, such as habitat structure, food- quantity and temperature (Crowder & Cooper, 1982). Effective predation also depends on predator hunting strategy and its flexibility (Grabowska et al., 2009).

Under experimental conditions *D. villosus* exhibited higher survival than other gammarids, including both native and invasive species, in the presence of diverse predators, such as the sympatric Ponto-Caspian gobies or the allopatric European bullhead and Amur sleeper (Kobak, Jermacz & Płąchocki, 2014; Błońska et al., 2015; Błońska et al., 2016; Beggel et al., 2016). Moreover, Kley et al. (2009) observed that the turbot (*Lota lota*) consumed fewer *D. villosus* compared to *Gammarus roeselii*. A similar result was shown by Błońska et al. (2015), who demonstrated that the racer goby always consumed preferentially native *G. fossarum* over *D. villosus* even if the gammarids were immobilized and unable to defend themselves. However, the goby did not exhibit any selectivity towards the waterborne chemical signals of native and invasive amphipods in a Y-maze (Błońska et al., 2015). On the other hand, Błońska et al. (2016) demonstrated that immobilized *D. villosus* and native *G. fossarum* were equally selected by other goby species (the round goby *Neogobius melanostomus* and the tubenose goby *Proterorhinus semilunaris*) and the European bullhead, whereas mobile *D. villosus* specimens were avoided, irrespective of the presence or absence of shelters. This indicates that the effective behavioural anti-predator responses of *D. villosus* determined its survival under the pressure of these predator species (Błońska et al., 2016). The coarse and complex substratum (gravel, stones and zebra mussel colonies) also improved the survival of *D. villosus* compared to fine substrata and other gammarid species (Kinzler & Maier, 2006; Kobak, Jermacz & Płąchocki, 2014). These results suggest that the mechanisms of the resistance of *D. villosus* to different predators may vary depending on their hunting mode, size and/or other traits.

The effectiveness of goby predation on *D. villosus* was described in detail by Jermacz et al. (2017). They demonstrated that under particular conditions, for example when gammarids were active or aggregated, the percentage of successful gobiid attacks was lower than 25%. The predation efficiency exceeded 50% only in the case of single inactive gammarid individuals. Moreover, even when a fish already had a gammarid in its mouth, the prey was still able to escape without any visible damage. Such a low effectiveness of predation forces predatory species to multiply their effort to achieve the desired satiation level or choose alternative prey species if available. The necessity of feeding on prey generating high handling costs is unfavourable for the predator condition. For example, under laboratory conditions Błońska et al. (2015) demonstrated that gobiids fed with native *G. fossarum* or chironomid larvae grew significantly better than individuals forced to feed on *D. villosus*, and the latter group of fish exhibited weight loss after a 4-week exposure.

These observations confirm that *D. villosus* is a comparatively poor food item for its potential predators, and is likely to be avoided in the presence of alternative prey species, which can make it relatively safe in the natural environment. Generally, amphipods are considered as one of the most important elements of the diet of many fish species (MacNeil, Dick & Elwood, 1999), however experimental results demonstrated that the role of *D. villosus* as food for the fish community could be significantly different from that of its native counterparts (Kobak, Jermacz & Płąchocki, 2014; Błońska et al., 2015; Błońska et al., 2016; Beggel et al., 2016), often replaced by the alien species (Dick & Platvoet, 2000; Dick, Platvoet & Kelly, 2002; Grabowski et al., 2006).

### Positive response of *D. villosus* to the predation cue

In general, a chemical signal indicating predator presence induces a defence response responsible for the reduction in predation risk (Brönmark & Hansson, 2000; Ferrari, Wisenden & Chivers, 2010). However, in the case of omnivorous species, capable of feeding on predator faeces or their dead bodies, or partly sharing their diet, a predation signal does not always indicate only a danger and, as a consequence, does not always induce a defence response. Such a unique situation takes place in the case of *D. villosus* as it actively avoided the scent of hungry predators in a Y-maze, but did not exhibit an avoidance reaction to the predation cues emitted by predators fed with chironomids or other gammarids (including conspecifics) (Jermacz, Dzierżyńska-Białończyk & Kobak, 2017). On the contrary, it showed an active preference, moving towards the scent of satiated predators in a Y-maze (Jermacz, Dzierżyńska-Białończyk & Kobak, 2017). A similar response was induced by the presence of cues released by crushed conspecifics and other gammarid species. This reaction suggests that this omnivorous and cannibalistic species is able to use such a signal not as a predation cue, but as a source of information about the location of a feeding ground. As shown in the above sections of this review, *D. villosus* is characterized by an effective defence strategy (Kobak, Jermacz & Płąchocki, 2014; Błońska et al., 2015; Błońska et al., 2016; Jermacz et al., 2017), therefore being relatively safe in the presence of predators, especially when alternative prey items are available in the vicinity (Jermacz et al., 2015a; Błońska et al., 2016). In such a situation, *D. villosus* may follow a predator to feed on its faeces or sense wounded invertebrates as being its potential prey. A similar trade-off between predator avoidance and foraging was observed in the case of *Gammarus pulex,* which in the presence of food did not respond to the predation signal, contrary to the situation when it was exposed only to predator kairomones (Szokoli et al., 2015).

### Costs of the anti-predator responses of *D. villosus*

Anti-predatory defences of prey organisms usually result in considerable energetic costs of the development of additional structures, selection of suboptimal habitats and/or decreased feeding due to the higher vigilance focused on predator detection (Hawlena & Schmitz, 2010; Sheriff & Thaler, 2014). This may result in growth reduction (Janssens & Stoks, 2013), weaker condition (Slos & Stoks, 2008) and/or finally even mortality (McCauley, Rowe & Fortin, 2011). The impact of the presence of predators on the feeding of *D. villosus* was checked by Jermacz & Kobak (2017). The gammarids considerably limited their feeding in the presence of predators (by 95% and 74% depending on the location of food, placed in the direct vicinity of shelters or away from them, respectively). Surprisingly, this response was even stronger than that of the related species *P. robustoides* (77% and 33%, respectively), though the latter seems to be more susceptible to predation pressure. On the other hand, no decrease in feeding was observed when single gammarids did not have to search for their food, having it available directly in their shelters. This shows that the aforementioned limitation of feeding in the open field resulted from the limited activity of gammarids (when food was located close to the shelter) or their increased vigilance in the open field (when food was distant from the shelter and no reduction in the search time was observed).

Nevertheless, the most important result of the study by Jermacz & Kobak (2017) was the demonstration that the growth rate of *D. villosus* supplied with food in their shelters (over a period of 2 weeks) was unaffected by the presence of predators. On the other hand, *P. robustoides* under the same conditions significantly reduced its growth rate by ca. 60% when exposed to predation cues (Jermacz & Kobak, 2017). Reduction in growth under predation risk was also observed in an amphipod *Hyalella azteca*, accompanying its induced morphological adaptations resulting in lower predator pressure (James & McClintock, 2017). This confirms the relatively high resistance of *D. villosus* to non-consumptive predator effects and shows that it may thrive in a good physiological condition under predatory pressure.

Resistance of *D. villosus* to predator non-consumptive effects was also confirmed by Richter et al. (2018), who did not observe any disturbance of gammarid feeding behaviour under the pressure of a benthivorous fish, the European bullhead (*Cottus gobio*). In contrast, another gammarid species (*G. pulex*) reduced its consumption in the presence of *C. gobio* kairomones (Abjörnsson et al., 2000). Lagrue, Besson & Lecerf (2015) have shown that the abundance of armoured detritivore prey did not decrease in the presence of predators, in contrast to that of non-armoured species. *D. villosus* is more armoured than other gammarids (Błońska et al., 2015), and thus the consequence of trade-offs between behavioural and morphological defences, such as the cost of the anti-predator responses of *D. villosus* seems to be less pronounced than that of other gammarids.

### Ecological significance of the anti-predator strategy of *D. villosus*

We have shown that *D. villosus* is capable of flexible predator recognition (Jermacz, Dzierżyńska-Białończyk & Kobak, 2017) allowing the species to respond to both novel and known dangers. It seems unlikely that it may benefit from the naïvety of local predators in central and western Europe, as they are used to preying on native gammarids (MacNeil, Dick & Elwood, 1999; MacNeil, Elwood & Dick, 1999) and not very selective with regard to their benthic food, consuming also large quantities of alien amphipods (Rezsu & Specziár, 2006; Eckmann et al., 2008). Moreover, predators of Ponto-Caspian origin, sympatric to the gammarids, such as several species of gobiid fish, have also invaded the same regions and co-occur with *D. villosus* in most of its current range, and include it in their diet (Grabowska & Grabowski, 2005; Borza, Eros & Oertel, 2009; Brandner et al., 2013). Therefore, its ability to easily recognize potential dangers may be one of the traits facilitating its establishment in invaded areas.

The efficient defence mechanisms of *D. villosus* make this species relatively resistant to predation (Kobak, Jermacz & Płąchocki, 2014; Jermacz et al., 2017; Jermacz, Dzierżyńska-Białończyk & Kobak, 2017), which may help it in its competition with other gammarids (Jermacz et al., 2015a; Beggel et al., 2016). Other, less resistant and more often consumed species are preferentially removed from the environment by predators and must spend more energy and time on anti-predator vigilance, whereas *D. villosus*, as the least preferred potential food, may thrive in the presence of predators with no negative effect on its growth (Jermacz & Kobak, 2017). Moreover, its aggressive behaviour may force competing gammarid species to less suitable habitats (Platvoet et al., 2009; Jermacz et al., 2015a) or make them swim more often in the water column, which further exposes them to fish predation (e.g., Jermacz et al., 2015a; Beggel et al., 2016). In addition to direct intra-guild predation and competition for food, this displacement from shelter is likely to be another factor making *D. villosus* an efficient competitor displacing other species from the areas in which it appears. Negative interactions with *D. villosus* make other gammarids avoid the presence of the stronger competitor, increasing their migrations to new areas and switching to different habitats (Dick, Platvoet & Kelly, 2002; Hesselschwerdt, Necker & Wantzen, 2008; Platvoet et al., 2009; Jermacz et al., 2015a; Kobak et al., 2016).

Nevertheless, the nature of interactions between *D. villosus* and its related species is far more complex. The presence of predators does have an impact on *D. villosus* behaviour (Jermacz, Dzierżyńska-Białończyk & Kobak, 2017; Kobak et al., 2017; Jermacz et al., 2017) and may reduce its interspecific aggression, allowing the competing species to stay in its presence. Jermacz et al. (2015a) have demonstrated that another gammarid *P. robustoides* is easily displaced from habitats preferred by both species in a safe environment, but the presence of predatory fish changes the situation, allowing *P. robustoides* to stay in the area co-occupied by *D. villosus*. It is difficult to distinguish whether this is due to the reduction in *D. villosus* aggression or the higher substratum affinity of *P. robustoides* in the presence of predators (selecting the vicinity of the stronger competitor as the lesser evil), or both. Nevertheless, individuals of both species can take advantage of staying in a group and reduce the probability of a successful predator attack (Jermacz et al., 2017). This also shows how important it is to consider the effect of predators when studying competitive interactions between species because the consequences of competition in a predator-free situation, which is very unlikely in the wild, may be easily overestimated.

Moreover, as the reduction in the feeding rate of *D. villosus* in the presence of predators was observed by Jermacz & Kobak (2017), it is likely that the predatory impact of this gammarid on the local community can also be lower than expected on the basis of experiments conducted in fishless conditions. This confirms the results of the field studies in the River Rhine (Hellmann et al., 2015; Hellmann et al., 2017; Koester, Bayer & Gergs, 2016) and can explain their discrepancy with some laboratory experiments, indicating the strong predatory impact of *D. villosus* on invertebrates (Dick & Platvoet, 2000; MacNeil & Platvoet, 2005).

Unexpectedly, despite the high consumption of *D. villosus* commonly observed in the field (Kelleher et al., 1998; Grabowska & Grabowski, 2005; Eckmann et al., 2008; Borza, Eros & Oertel, 2009; Brandner et al., 2013; Czarnecka, Pilotto & Pusch, 2014), it was experimentally demonstrated that its dominance may in fact decrease the quality of food conditions for fish due to the higher difficulty of capturing and handling, leading to poor growth on a diet based on this species, compared to the diets consisting of native gammarids or chironomid larvae (Błońska et al., 2015). Thus, although fish feed on *D. villosus* in the areas invaded by this species, it seems they would have thrived much better if this invasion had not occurred and other gammarid species (usually displaced by the invader) had been available as alternative food (Błońska et al., 2015).

## Conclusion

We have shown *D. villosus* as a species with efficient anti-predation mechanisms (both behavioural modifications and constitutive traits), relatively safe from predators and bearing lower costs of their non-consumptive effects (as indicated by its growth unaffected by the presence of fish), compared to related taxa. It can recognize sympatric and novel fish predators independent of their diet, though its precise responses are fine-tuned on the basis of food consumed by a predator, and can range from avoidance to preference. Sometimes *D. villosus* can even be attracted to a predator scent, probably utilizing their presence to locate potential food sources. Defence mechanisms of this species include activity reduction, aggregation and migration. In general, single immobile individuals outside the shelter are the most susceptible to predation. Therefore, threatened individuals try: (1) to stay in the shelter, at best co-occupied by other specimens; (2) if this is not possible, to move in search of a shelter; (3) if shelters are difficult to find, to aggregate with conspecifics, used as a substitute shelter; (4) if conspecifics are also difficult to locate (e.g., at a low density), to relocate to safer areas, e.g., away from the predator scent or to the shallower bottom. These traits are likely to give it a strong advantage in competition with similar species, both natives and other invaders, and contribute to its invasive potential. Moreover, we have demonstrated the strong importance of predator effects on interactions among gammarid species involving *D. villosus*, which cannot be neglected in future studies on this topic. It is likely that under predatory pressure the competitive impact of *D. villosus* on other gammarids as well as its predation on zoobenthos organisms are reduced, altering its impact on local communities.

## Additional Information and Declarations

### Data Availability

The following information was supplied regarding data availability: The research in this article did not generate any data or code, this is a comprehensive literature review of the responses of an invasive amphipod *Dikerogammarus villosus* to predator signal.

## References

[ref-1] Abjörnsson K, Dahl J, Nyström P, Brönmark C (2000). Influence of predator and dietary chemical cues on the behaviour and shredding efficiency of *Gammarus pulex*. Aquatic Ecology.

[ref-2] Åbjörnsson K, Hansson L-A, Brönmark C (2004). Responses of prey from habitats with different predator regimes: local adaptation and heritability. Ecology.

[ref-3] Åbjörnsson K, Wagner B, Axelsson A, Bjerselius R, Olsen KH (1997). Responses of *Acilius sulcatus* (Coleoptera: Dytiscidae) to chemical cues from perch (*Perca fluviatilis*). Oecologia.

[ref-4] Andersson KG, Brönmark C, Herrmann J, Malmqvist B, Otto C, Sjörström P, Bronmark C (1986). Presence of sculpins (*Cottus gobio*) reduces drift and activity of *Gammarus pulex* (Amphipoda). Hydrobiologia.

[ref-5] Bacela-Spychalska K (2016). Attachment ability of two invasive amphipod species may promote their spread by overland transport. Aquatic Conservation: Marine and Freshwater Ecosystems.

[ref-6] Bacela-Spychalska K, Grabowski M, Rewicz T, Konopacka A, Wattier R (2013). The ‘killer shrimp’ *Dikerogammarus villosus* (Crustacea, Amphipoda) invading Alpine lakes: overland transport by recreational boats and scuba-diving gear as potential entry vectors?. Aquatic Conservation: Marine and Freshwater Ecosystems.

[ref-7] Bacela-Spychalska K, Rigaud T, Wattier RA (2014). A co-invasive microsporidian parasite that reduces the predatory behaviour of its host *Dikerogammarus villosus* (Crustacea, Amphipoda). Parasitology.

[ref-8] Bacela-Spychalska K, Wattier RA, Genton C, Rigaud T (2012). Microsporidian disease of the invasive amphipod *Dikerogammarus villosus* and the potential for its transfer to local invertebrate fauna. Biological Invasions.

[ref-9] Banks PB, Dickman CR (2007). Alien predation and the effects of multiple levels of prey naiveté. Trends in Ecology & Evolution.

[ref-10] Becker J, Ortmann C, Wetzel MA, Koop JHE (2016). Metabolic activity and behavior of the invasive amphipod *Dikerogammarus villosus* and two common Central European gammarid species (*Gammarus fossarum*, *Gammarus roeselii*): low metabolic rates may favor the invader. Comparative Biochemistry and Physiology—Part A: Molecular and Integrative Physiology.

[ref-11] Beekey MA, McCabe DJ, Marsden JE (2004). Zebra mussels affect benthic predator foraging success and habitat choice on soft sediments. Oecologia.

[ref-12] Beggel S, Brandner J, Cerwenka AF, Geist J (2016). Synergistic impacts by an invasive amphipod and an invasive fish explain native gammarid extinction. BMC Ecology.

[ref-13] Bij de Vaate A, Jazdzewski K, Ketelaars HAM, Gollasch S, Van der Velde G (2002). Geographical patterns in range extension of Ponto-Caspian macroinvertebrate species in Europe. Canadian Journal of Fisheries and Aquatic Sciences.

[ref-14] Błońska D, Grabowska J, Kobak J, Jermacz Ł, Bącela-Spychalska K (2015). Feeding preferences of an invasive Ponto-Caspian goby for native and non-native gammarid prey. Freshwater Biology.

[ref-15] Błońska D, Grabowska J, Kobak J, Rachalewski M, Bącela-Spychalska K (2016). Fish predation on sympatric and allopatric prey—a case study of Ponto-Caspian gobies, European bullhead and amphipods. Limnologica—Ecology and Management of Inland Waters.

[ref-16] Boeing WJ, Ramcharan CW, Riessen HP (2006). Clonal variation in depth distribution of *Daphnia pulex* in response to predator kairomones. Archiv für Hydrobiologie.

[ref-17] Boets P, Lock K, Messiaen M, Goethals PLM (2010). Combining data-driven methods and lab studies to analyse the ecology of *Dikerogammarus villosus*. Ecological Informatics.

[ref-18] Bollache L, Kaldonski N, Troussard J-P, Lagrue C, Rigaud T (2006). Spines and behaviour as defences against fish predators in an invasive freshwater amphipod. Animal Behaviour.

[ref-19] Borza P, Eros T, Oertel N (2009). Food resource partitioning between two invasive gobiid species (Pisces, Gobiidae) in the littoral zone of the river danube, Hungary. International Review of Hydrobiology.

[ref-20] Brandner J, Auerswald K, Cerwenka AF, Schliewen UK, Geist J (2013). Comparative feeding ecology of invasive Ponto-Caspian gobies. Hydrobiologia.

[ref-21] Brönmark C, Hansson L (2000). Chemical communication in aquatic systems: an introduction. Oikos.

[ref-22] Chivers DP, Smith RJF (1998). Chemical alarm signalling in aquatic predator-prey systems: a review and prospectus. Ecoscience.

[ref-23] Chivers DP, Wisenden BD, Smith RJF (1996). Damselfly larvae learn to recognize predators from chemical cues in the predator’s diet. Animal Behaviour.

[ref-24] Clinchy M, Sheriff MJ, Zanette LY (2013). Predator-induced stress and the ecology of fear. Functional Ecology.

[ref-25] Creel S, Christianson D (2008). Relationships between direct predation and risk effects. Trends in Ecology and Evolution.

[ref-26] Crowder LB, Cooper WE (1982). Habitat structural complexity and the interaction between bluegills and their prey. Ecology.

[ref-27] Czarnecka M (2016). Coarse woody debris in temperate littoral zones: implications for biodiversity, food webs and lake management. Hydrobiologia.

[ref-28] Czarnecka M, Pilotto F, Pusch MT (2014). Is coarse woody debris in lakes a refuge or a trap for benthic invertebrates exposed to fish predation?. Freshwater Biology.

[ref-29] Czarnecka M, Poznańska M, Kobak J, Wolnomiejski N (2009). The role of solid waste materials as habitats for macroinvertebrates in a lowland dam reservoir. Hydrobiologia.

[ref-30] Czarnołęski M, Müller T, Adamus K, Ogorzelska G, Sog M (2010). Injured conspecifics alter mobility and byssus production in zebra mussels *Dreissena polymorpha*. Fundamental and Applied Limnology/Archiv für Hydrobiologie.

[ref-31] DAISIE (2009). Handbook of Alien Species in Europe.

[ref-32] Dalesman S, Rundle SD, Cotton PA (2007). Predator regime influences innate anti-predator behaviour in the freshwater gastropod *Lymnaea stagnalis*. Freshwater Biology.

[ref-33] De Meester L, Dawidowicz P, Loose C, Van Gool E, Tollrian R, Harvel CD (1999). Ecology and evolution of predator-induced behavior of zooplankton: depth selection behavior and diel vertical migration. The ecoloy and evolution of inducible defenses.

[ref-34] Devin S, Beisel JN (2007). Biological and ecological characteristics of invasive species: a gammarid study. Biological Invasions.

[ref-35] Devin S, Piscart C, Beisel JN, Moreteau JC (2003). Ecological traits of the amphipod invader *Dikerogammarus villosus* on a mesohabitat scale. Archiv für Hydrobiologie.

[ref-36] Dick JTA, Platvoet D (2000). Invading predatory crustacean *Dikerogammarus villosus* eliminates both native and exotic species. Proceedings of the Royal Society B: Biological Sciences.

[ref-37] Dick JTA, Platvoet D, Kelly DW (2002). Predatory impact of the freshwater invader *Dikerogammarus villosus* (Crustacea: Amphipoda). Canadian Journal of Fisheries and Aquatic Sciences.

[ref-38] Dunn AM, Dick JTA, Hatcher MJ (2008). The less amorous Gammarus: predation risk affects mating decisions in *Gammarus duebeni* (Amphipoda). Animal Behaviour.

[ref-39] Dzialowski AR, Lennon JT, O’Brien WJ, Smith VH (2003). Predator-induced phenotypic plasticity in the exotic cladoceran *Daphnia lumholtzi*. Freshwater Biology.

[ref-40] Eckmann R, Mörtl M, Baumgärtner D, Berron C, Fischer P, Schleuter D, Weber A (2008). Consumption of amphipods by littoral fish after the replacement of native *Gammarus roeseli* by invasive *Dikerogammarus villosus* in Lake Constance. Aquatic Invasions.

[ref-41] Emlen JM (1966). The role of time and energy in food preference. The American Naturalist.

[ref-42] Ferincz Á, Staszny Á, Weiperth A, Takács P, Urbányi B, Vilizzi L, Paulovits G, Copp GH (2016). Risk assessment of non-native fishes in the catchment of the largest Central-European shallow lake (Lake Balaton, Hungary). Hydrobiologia.

[ref-43] Ferrari MCO, Gonzalo A, Messier F, Chivers DP (2007). Generalization of learned predator recognition: an experimental test and framework for future studies. Proceedings of the Royal Society B: Biological Sciences.

[ref-44] Ferrari MCO, Wisenden BD, Chivers DP (2010). Chemical ecology of predator—prey interactions in aquatic ecosystems: a review and prospectus. The present review is one in the special series of reviews on animal-plant interactions. Canadian Journal of Zoology.

[ref-45] Gergs R, Rothhaupt K-O (2008a). Effects of zebra mussels on a native amphipod and the invasive *Dikerogammarus villosus*: the influence of biodeposition and structural complexity. Journal of North American Benthological Society.

[ref-46] Gergs R, Rothhaupt K-O (2008b). Feeding rates, assimilation efficiencies and growth of two amphipod species on biodeposited material from zebra mussels. Freshwater Biology.

[ref-47] Gergs R, Rothhaupt K-O (2015). Invasive species as driving factors for the structure of benthic communities in Lake Constance, Germany. Hydrobiologia.

[ref-48] Gerking SD (1994). Feeding ecology of fish.

[ref-49] Glazier DS, Butler EM, Lombardi SA, Deptola TJ, Reese AJ, Satterthwaite EV (2011). Ecological effects on metabolic scaling: Amphipod responses to fish predators in freshwater springs. Ecological Monographs.

[ref-50] Gliwicz ZM (1994). Relative significance of direct and indirect effects of predation by planktivorous fish on zooplankton. Hydrobiologia.

[ref-51] Gliwicz ZM (2005). Food web interactions: why are they reluctant to be manipulated? Plenary Lecture. Verhandlungen Internationale Vereinigung für Theoretische und Angewandte Limnologie.

[ref-52] Gliwicz ZM, Słoń J, Szynkarczyk I (2006). Trading safety for food: evidence from gut contents in roach and bleak captured at different distances offshore from their daytime littoral refuge. Freshwater Biology.

[ref-53] Grabowska J, Grabowski M (2005). Diel-feeding activity in early summer of racer goby *Neogobius gymnotrachelus* (Gobiidae): a new invader in the Baltic basin. Journal of Applied Ichthyology.

[ref-54] Grabowska J, Grabowski M, Pietraszewski D, Gmur J (2009). Non-selective predator—the versatile diet of Amur sleeper (*Perccottus glenii* Dybowski, 1877) in the Vistula River (Poland), a newly invaded ecosystem. Journal of Applied Ichthyology.

[ref-55] Grabowski M, Bącela K, Konopacka A (2007). How to be an invasive gammarid (Amphipoda: Gammaroidea)—comparison of life history traits. Hydrobiologia.

[ref-56] Grabowski M, Konopacka A, Jazdzewski K, Janowska E (2006). Invasions of alien gammarid species and retreat of natives in the Vistula Lagoon (Baltic Sea, Poland). Helgoland Marine Research.

[ref-57] Gusev AA, Guseva DO, Sudnik SA (2017). New record of the Ponto-Caspian gammarid *Dikerogammarus villosus* (Sowinsky, 1894) in the southeastern part of the Baltic Sea (Kaliningrad oblast, Russia). Russian Journal of Biological Invasions.

[ref-58] Hamilton WD (1971). Geometry for the selfish herd. Journal of Theoretical Biology.

[ref-59] Hawlena D, Schmitz OJ (2010). Physiological stress as a fundamental mechanism linking predation to ecosystem functioning. The American Naturalist.

[ref-60] Hellmann C, Schöll F, Worischka S, Becker J, Winkelmann C (2017). River-specific effects of the invasive amphipod *Dikerogammarus villosus* (Crustacea: Amphipoda) on benthic communities. Biological Invasions.

[ref-61] Hellmann C, Worischka S, Mehler E, Becker J, Gergs R, Winkelmann C (2015). The trophic function of *Dikerogammarus villosus* (Sowinsky, 1894) in invaded rivers: a case study in the Elbe and Rhine. Aquatic Invasions.

[ref-62] Hesselschwerdt J, Necker J, Wantzen KM (2008). Gammarids in Lake Constance: habitat segregation between the invasive *Dikerogammarus villosus* and the indigenous *Gammarus roeselii*. Fundamental and Applied Limnology/Archiv für Hydrobiologie.

[ref-63] Hesselschwerdt J, Tscharner S, Necker J, Wantzen KM (2009). A local gammarid uses kairomones to avoid predation by the invasive crustaceans *Dikerogammarus villosus* and *Orconectes limosus*. Biological Invasions.

[ref-64] Holomuzki JR, Hoyle JD (1988). Effect of predatory fish presence on habitat use and diel movement of the stream amphipod, *Gammarus minus*. Freshwater Biology.

[ref-65] Ioannou CC, Krause J (2008). Searching for prey: the effects of group size and number. Animal Behaviour.

[ref-66] James WR, McClintock JB (2017). Anti-predator responses of amphipods are more effective in the presence of conspecific chemical cues. Hydrobiologia.

[ref-67] Janssens L, Stoks R (2013). Predation risk causes oxidative damage in prey. Biology Letters.

[ref-68] Jermacz Ł, Andrzejczak J, Arczyńska E, Zielska J, Kobak J (2017). An enemy of your enemy is your friend: impact of predators on aggregation behavior of gammarids. Ethology.

[ref-69] Jermacz Ł, Dzierzyńska A, Kakareko T, Poznańska M, Kobak J (2015a). The art of choice: predation risk changes interspecific competition between freshwater amphipods. Behavioral Ecology.

[ref-70] Jermacz Ł, Dzierzyńska A, Poznańska M, Kobak J (2015b). Experimental evaluation of preferences of an invasive Ponto-Caspian gammarid *Pontogammarus robustoides* (Amphipoda, Gammaroidea) for mineral and plant substrata. Hydrobiologia.

[ref-71] Jermacz Ł, Dzierżyńska-Białończyk A, Kobak J (2017). Predator diet, origin or both? Factors determining responses of omnivorous amphipods to predation cues. Hydrobiologia.

[ref-72] Jermacz Ł, Kobak J (2017). Keep calm and don’t stop growing: non-consumptive effects of a sympatric predator on two invasive Ponto-Caspian gammarids *Dikerogammarus villosus* and *Pontogammarus robustoides*. PLOS ONE.

[ref-73] Kaldonski N, Perrot-Minnot M-J, Motreuil S, Cézilly F (2008). Infection with acanthocephalans increases the vulnerability of *Gammarus pulex* (Crustacea, Amphipoda) to non-host invertebrate predators. Parasitology.

[ref-74] Kelleher B, Bergers PJM, Van den Brink FWB, Giller PS, Van der Velde G, De Vaate AB (1998). Effects of exotic amphipod invasions on fish diet in the Lower Rhine. Fundamental and Applied Limnology.

[ref-75] Kinzler W, Kley A, Mayer G, Waloszek D, Maier G (2009). Mutual predation between and cannibalism within several freshwater gammarids: *Dikerogammarus villosus* versus one native and three invasives. Aquatic Ecology.

[ref-76] Kinzler W, Maier G (2006). Selective predation by fish: a further reason for the decline of native gammarids in the presence of invasives?. Journal of Limnology.

[ref-77] Kley A, Kinzler W, Schank Y, Mayer G, Waloszek D, Maier G (2009). Influence of substrate preference and complexity on co-existence of two non-native gammarideans (Crustacea: Amphipoda). Aquatic Ecology.

[ref-78] Kobak J, Jermacz Ł, Dzierżyńska-Białończyk A (2015). Substratum preferences of the invasive killer shrimp *Dikerogammarus villosus*. Journal of Zoology.

[ref-79] Kobak J, Jermacz Ł, Płąchocki D (2014). Effectiveness of zebra mussels to act as shelters from fish predators differs between native and invasive amphipod prey. Aquatic Ecology.

[ref-80] Kobak J, Jermacz Ł, Rutkowska D, Pawłowska K, Witkowska L, Poznańska M (2017). Impact of predators and competitors on the depth selection by two invasive gammarids. Journal of Zoology.

[ref-81] Kobak J, Kakareko T, Poznańska M (2010). Changes in attachment strength and aggregation of zebra mussel, *Dreissena polymorpha* in the presence of potential fish predators of various species and size. Hydrobiologia.

[ref-82] Kobak J, Poznańska M, Jermacz Ł, Kakareko T, Prądzynski D, Łodygowska M, Montowska K, Bącela-Spychalska K (2016). Zebra mussel beds: an effective feeding ground for Ponto-Caspian gobies or suitable shelter for their prey?. PeerJ.

[ref-83] Kobak J, Rachalewski M, Bącela-Spychalska K (2016). Conquerors or exiles? Impact of interference competition among invasive Ponto-Caspian gammarideans on their dispersal rates. Biological Invasions.

[ref-84] Kobak J, Ryńska A (2014). Environmental factors affecting behavioural responses of an invasive bivalve to conspecific alarm cues. Animal Behaviour.

[ref-85] Koester M, Bayer B, Gergs R (2016). Is *Dikerogammarus villosus* (Crustacea, Gammaridae) a ‘killer shrimp’ in the River Rhine system?. Hydrobiologia.

[ref-86] Koperski P (1997). Changes in feeding behaviour of the larvae of the damselfly *Enallagma cyathigerum* in response to stimuli from predators. Ecological Entomology.

[ref-87] Korpi NL, Wisenden BD (2001). Learned recognition of novel predator odour by zebra danios, *Danio rerio*, Following time-shifted presentation of alarm cue and predator odour. Environmental Biology of Fishes.

[ref-88] Krisp H, Maier G (2005). Consumption of macroinvertebrates by invasive and native gammarids: a comparison. Journal of Limnology.

[ref-89] Lafferty KD, Morris AK (1996). Altered behavior of parasitized killifish increases susceptibility to predation by bird final hosts. Ecology.

[ref-90] Lagrue C, Besson AA, Lecerf A (2015). Interspecific differences in antipredator strategies determine the strength of non-consumptive predator effects on stream detritivores. Oikos.

[ref-91] Lass S, Spaak P (2003). Chemically induced anti-predator defences in plankton: a review. Hydrobiologia.

[ref-92] Lima SLS, Dill LML (1990). Behavioral decisions made under the risk of predation: a review and prospectus. Canadian Journal of Zoology.

[ref-93] Maazouzi C, Piscart C, Pihan J-C, Masson G (2009). Effect of habitat-related resources on fatty acid composition and body weight of the invasive *Dikerogammarus villosus* in an artificial reservoir. Fundamental and Applied Limnology/Archiv für Hydrobiologie.

[ref-94] MacNeil C, Dick JTA, Elwood RW (1999). The dynamics of predation on Gammarus spp. (Crustacea: Amphipoda). Biological Reviews.

[ref-95] MacNeil C, Dick JTA, Platvoet D, Briffa M (2011). Direct and indirect effects of species displacements: an invading freshwater amphipod can disrupt leaf-litter processing and shredder efficiency. Journal of the North American Benthological Society.

[ref-96] MacNeil C, Elwood RW, Dick JTA (1999). Predator-prey interactions between brown trout *Salmo trutta* and native and introduced ampbipods; tbeir implications for fish diets. Ecography.

[ref-97] MacNeil C, Platvoet D (2005). The predatory impact of the freshwater invader *Dikerogammarus villosus* on native *Gammarus pulex* (Crustacea: Amphipoda); influences of differential microdistribution and food resources. Journal of Zoology.

[ref-98] Martens A, Grabow K (2008). Das Risiko der Verschleppung neozoischer Amphipoda beim Uberlandtransport von Yachten. Lauterbornia.

[ref-99] Martin CW (2014). Naïve prey exhibit reduced antipredator behavior and survivorship. PeerJ.

[ref-100] Mastitsky SE, Makarevich OA (2007). Distribution and abundance of Ponto-Caspian amphipods in the Belarusian section of the Dnieper River. Aquatic Invasions.

[ref-101] Mayer G, Maas A, Waloszek D (2012). Coexisting native and non-indigenous gammarideans in lake constance-comparative morphology of mouthparts (crustacea, amphipoda, gammaridea). Spixiana.

[ref-102] McCabe DJ, Beekey MA, Mazloff A, Marsden JE (2006). Negative effect of zebra mussels on foraging and habitat use by lake sturgeon (*Acipenser fulvescens*). Aquatic Conservation: Marine and Freshwater Ecosystems.

[ref-103] McCauley SJ, Rowe L, Fortin M-J (2011). The deadly effects of “nonlethal” predators. Ecology.

[ref-104] Meijer K, Schilthuizen M, Beukeboom L, Smit C (2016). A review and meta-analysis of the enemy release hypothesis in plant-herbivorous insect systems. PeerJ.

[ref-105] Mowles SL, Rundle SD, Cotton PA (2011). Susceptibility to predation affects trait-mediated indirect interactions by reversing interspecific competition. PLOS ONE.

[ref-106] Muskó IB, Balogh C, Tóth ÁP, Varga É, Lakatos G (2007). Differential response of invasive malacostracan species to lake level fluctuations. Hydrobiologia.

[ref-107] Pennuto C, Keppler D (2008). Short-term predator avoidance behavior by invasive and native amphipods in the Great Lakes. Aquatic Ecology.

[ref-108] Perez KO, Carlson RL, Shulman MJ, Ellis JC (2009). Why are intertidal snails rare in the subtidal? Predation, growth and the vertical distribution of *Littorina littorea* (L.) in the Gulf of Maine. Journal of Experimental Marine Biology and Ecology.

[ref-109] Perrot-Minnot MJ, Kaldonski N, Cézilly F (2007). Increased susceptibility to predation and altered anti-predator behaviour in an acanthocephalan-infected amphipod. International Journal for Parasitology.

[ref-110] Pettersson LB, Nilsson PA, Brönmark C (2000). Predator recognition and defence strategies in crucian carp, *Carassius carassius*. Oikos.

[ref-111] Pinchuk VI, Vasileva ED, Vasilev VP, Miller P, Miller P (2003). *Neogobius gymnotrachelus* (Kessler, 1857). The freshwater fishes of Europe, Vol. 8. I Mugilidae, Atherinidae, Atherinopsidae, Blenniidae, Odontobutidae, Gobiidae 1.

[ref-112] Platvoet D, Dick JTA, MacNeil C, Van Riel MC, Van der Velde G (2009). Invader–invader interactions in relation to environmental heterogeneity leads to zonation of two invasive amphipods, *Dikerogammarus villosus* (Sowinsky) and *Gammarus tigrinus* Sexton: amphipod pilot species project (AMPIS) report 6. Biological Invasions.

[ref-113] Poznańska M, Kakareko T, Krzyzyński M, Kobak J (2013). Effect of substratum drying on the survival and migrations of Ponto-Caspian and native gammarids (Crustacea: Amphipoda). Hydrobiologia.

[ref-114] Preisser EL, Bolnick DI, Benard MF (2005). Scared to death? The effects of intimidation and consumption in predator-prey interactions. Ecology.

[ref-115] Rewicz T, Grabowski M, MacNeil C, Bącela-Spychalska K (2014). The profile of a ‘perfect’ invader—the case of killer shrimp, *Dikerogammarus villosus*. Aquatic Invasions.

[ref-116] Rewicz T, Wattier R, Rigaud T, Grabowski M, Mamos T, Bącela-Spychalska K (2017). The killer shrimp, *Dikerogammarus villosus*, invading European Alpine Lakes: a single main source but independent founder events with an overall loss of genetic diversity. Freshwater Biology.

[ref-117] Rezsu E, Specziár A (2006). Ontogenetic diet profiles and size-dependent diet partitioning of ruffe *Gymnocephalus cernuus*, perch *Perca fluviatilis* and pumpkinseed *Lepomis gibbosus* in Lake Balaton. Ecology of Freshwater Fish.

[ref-118] Richter L, Schwenkmezger L, Becker J, Winkelmann C, Hellmann C, Worischka S (2018). The very hungry amphipod: the invasive *Dikerogammarus villosus* shows high consumption rates for two food sources and independent of predator cues. Biological Invasions.

[ref-119] Salo P, Korpimaki E, Banks PB, Nordstrom M, Dickman CR (2007). Alien predators are more dangerous than native predators to prey populations. Proceedings of the Royal Society B: Biological Sciences.

[ref-120] Sheriff MJ, Thaler JS (2014). Ecophysiological effects of predation risk; an integration across disciplines. Oecologia.

[ref-121] Šidagyte E, Solovjova S, Šniaukštaite V, Šiaulys A, Olenin S, Arbačiauskas K (2017). The killer shrimp *Dikerogammarus villosus* (Crustacea, Amphipoda) invades Lithuanian waters, South-Eastern Baltic Sea. Oceanologia.

[ref-122] Sih A, Bolnick DI, Luttbeg B, Orrock JL, Peacor SD, Pintor LM, Preisser E, Rehage JS, Vonesh JR (2010). Predator-prey naïveté, antipredator behavior, and the ecology of predator invasions. Oikos.

[ref-123] Slos S, Stoks R (2008). Predation risk induces stress proteins and reduces antioxidant defense. Functional Ecology.

[ref-124] Ślusarczyk M, Dawidowicz P, Rygielska E (2005). Hide, rest or die: a light-mediated diapause response in *Daphnia magna* to the threat of fish predation. Freshwater Biology.

[ref-125] Ślusarczyk M, Rygielska E (2004). Fish faeces as the primary source of chemical cues inducing fish avoidance diapause in *Daphnia magna*. Hydrobiologia.

[ref-126] Sornom P, Gismondi E, Vellinger C, Devin S, Férard J-F, Beisel J-N (2012). Effects of sublethal cadmium exposure on antipredator behavioural and antitoxic responses in the invasive amphipod *Dikerogammarus villosus*. PLOS ONE.

[ref-127] Szokoli F, Winkelmann C, Berendonk TU, Worischka S (2015). The effects of fish kairomones and food availability on the predator avoidance behaviour of *Gammarus pulex*. Fundamental and Applied Limnology.

[ref-128] Thoms C, Schupp PJ, Custódio MR, Lôbo-Hajdu G, Hajdu E, Muricy G (2007). Chemical defense strategies in sponges: a review. Porifera Research: Biodiversity, Innovation and Sustainability.

[ref-129] Torchin ME, Lafferty KD, Dobson AP, McKenzie VJ, Kuris AM (2003). Introduced species and their missing parasites. Nature.

[ref-130] Turner AM, Peacor SD (2012). Scaling up infochemicals. Chemical ecology in aquatic systems.

[ref-131] Ueshima E, Yusa Y (2015). Antipredator behaviour in response to single or combined predator cues in the apple snail *Pomacea canaliculata*. Journal of Molluscan Studies.

[ref-132] Van Riel MC, Healy EP, Van der Velde G, Bij de Vaate A (2007). Interference competition among native and invader amphipods. Acta Oecologica.

[ref-133] Van Riel MC, Van der Velde G, Rajagopal S, Marguillier S, Dehairs F, Bij de Vaate A (2006). Trophic relationships in the Rhine food web during invasion and after establishment of the Ponto-Caspian invader *Dikerogammarus villosus*. Hydrobiologia.

[ref-134] Von Elert E, Pohnert G (2000). Predator specificity of kairomones in diel vertical migration of Daphnia: a chemical approach. Oikos.

[ref-135] Weber A (2003). More than one “fish kairomone”? Perch and stickleback kairomones affect Daphnia life history traits differently. Hydrobiologia.

[ref-136] Werner EE, Peacor SD (2003). A review of trait-mediated indirect interactions in ecological communities. Ecology.

[ref-137] Wia̧ckowski K, Fyda J, Ciećko A (2004). The behaviour of an omnivorous protozoan affects the extent of induced morphological defence in a protozoan prey. Freshwater Biology.

[ref-138] Wisenden BD, Chivers DP, Smith RJF (1997). Learned recognition of predation risk by Enallagma damselfly larvae (Odonata, Zygoptera) on the basis of chemical cues. Journal of Chemical Ecology.

[ref-139] Wisenden BD, Rugg ML, Korpi NL, Fuselier LC (2009). Lab and field estimates of active time of chemical alarm cues of a cyprinid fish and an amphipod crustacean. Behaviour.

[ref-140] Wooster DE (1998). Amphipod (*Gammarus minus*) responses to predators and predator impact on amphipod density. Oecologia.

[ref-141] Wootton RJ (1990). Ecology of teleost fishes.

[ref-142] Wudkevich K, Wisenden BD, Chivers DP, Smith RJF (1997). Reactions of *Gammarus lacustris* to chemical stimuli from natural predators and injured conspecifics. Journal of Chemical Ecology.

[ref-143] Zuharah WF, Lester PJ (2010). Are exotic invaders less susceptible to native predators? A test using native and exotic mosquito species in New Zealand. Population Ecology.

